# Development of epithelial tissues: How are cleavage planes chosen?

**DOI:** 10.1371/journal.pone.0205834

**Published:** 2018-11-07

**Authors:** Ying Xin, Chathuri Madubhashini Karunarathna Mudiyanselage, Winfried Just

**Affiliations:** 1 Department of Mathematics, Ohio University, Athens, Ohio, 45701, United States of America; 2 Department of Mathematical Sciences, Montana State University, Bozeman, Montana, 59717, United States of America; 3 Quantitative Biology Institute, Ohio University, Athens, Ohio, 45701, United States of America; Laboratoire Arago, FRANCE

## Abstract

The cross-section of a cell in a monolayer epithelial tissue can be modeled mathematically as a *k*-sided polygon. Empirically studied distributions of the proportions of *k*-sided cells in epithelia show remarkable similarities in a wide range of evolutionarily distant organisms. A variety of mathematical models have been proposed for explaining this phenomenon. The highly parsimonious simulation model of (Patel et al., PLoS Comput. Biol., 2009) that takes into account only the number of sides of a given cell and cell division already achieves a remarkably good fit with empirical distributions from *Drosophila*, *Hydra*, *Xenopus*, Cucumber, and *Anagallis*. Within the same modeling framework as in that paper, we introduce additional options for the choice of the endpoints of the cleavage plane that appear to be biologically more realistic. By taking the same data sets as our benchmarks, we found that combinations of some of our new options consistently gave better fits with each of these data sets than previously studied ones. Both our algorithm and simulation data are made available as research tools for future investigations.

## Introduction

Epithelia are sheets of tightly adherent cells that line both internal and external surfaces in a vast array of metazoans. From the mathematical perspective, a (cross-section of a) cell in an epithelial tissue can be modeled as a *k*-sided polygon. The distributions of cellular polygons have been measured in a wide range of divergent organisms, both animal and plant, and are remarkably similar within select metazoan epithelia (differing by only a few percent), and are also similar between certain metazoans and some plant epidermis [1].

A variety of mathematical models that aim at explaining this phenomenon have been proposed in the literature. One class of models considers exclusively *cell topology*, that is the neighborhood relation between cells in the tissue. We will refer to them as *topological models*. The other class of models, called here *geometric models*, considers also such geometric features as size and shape of the polygon and permits the study of factors like the role of mechanical stresses. Another major distinction is between *division-only models* that consider only cell division as the mechanism for modification of tissue development, and models that also take into account such processes as cell rearrangements and apoptosis.

The simplest models are topological division-only ones. Prominent among them are the Markov chain model of [2], together with the related simulation-based follow-up paper [3]. Precursors of [2] include [4–7]. The papers [8, 9] contain improved versions of the Markov chain model, and [10–12] study simulation models similar to the one of [3]. We will briefly review a selection of other types of models in the Discussion section. For now let us only remark that development tends to occur in distinct stages [13], and processes other than cell division, in particular cell rearrangements, may play a significant role only at later stages that follow an earlier phase of proliferation where cell division dominates [2, 14, 15]. Thus one might expect division-only models to be more applicable to earlier developmental stages than to later ones.

Topological division-only models such as the one of [3] already give a remarkably good fit with what is considered in the literature by consensus the “standard polygonal distribution.” It was first pointed out in [2] that this distribution may simply be an emergent property of a stochastic process under very minimal assumptions about the underlying biological processes. Thus models like the ones of [2, 3] and ours may be considered as a default explanation, similar to a null hypothesis in statistics, with which more elaborate and detailed models might be compared. In the case of “nonstandard distributions” like the ones found in plants *Anacharis, Volvox* [7] and from later developmental stages of the chick embryo [9] these default models give a poor fit, so that a better fit obtained from a more detailed geometric model would indicate that the additional features or mechanisms accounted for in that model may explain the particular experimentally observed distribution. However, if a more detailed model does not significantly improve the fit relative to the best topological division-only model, there would be no compelling statistical evidence for drawing a similar conclusion.

In order to make such comparisons between a given model and an entire class of default models, one needs to know how good a fit can actually be achieved within that class. Thus we were interested in how much the fit for the model of [3] could be improved by considering more biologically plausible distributions for the endpoints of the cleavage plane and/or by modifying the division order. In order to assess the performance of these new options, we took as our benchmark the exact same sets of empirical data that were considered in [2, 3] and that exhibit standard polygonal distributions. We found that some of our new options for choosing the cleavage plane did give better fits than the model of [3]. This pattern was consistent for all five data sets. In contrast, the alternative division order studied by us usually did not result in improved fit. Our algorithm and simulation data can be used as research tools for comparison with additional data sets on early-stage epithelial development, including ones that may become available in the future.

## Materials and methods

### Assumptions of our model

We treat monolayer epithelial cells as *k*-sided polygons, where *k* is the number of neighbors of a cell. The following assumptions of [3] are made in our model:

1. The epithelial cell network is only modified by cell division. We do not consider any junctional rearrangements due to cell repacking, cell migration, or cell death.
2. A parent cell divides into two daughter cells through the creation of two trivalent vertices and one edge along the chosen cleavage plane. Thus daughter cells always share an edge.
3. When a cell divides, its cleavage plane must intersect two non-adjacent edges of the original cell. This precludes the formation of tetravalent vertices and 3-sided cells, both of which are rarely observed empirically.
4. Each cell divides exactly once per division cycle and the cells divide successively.

Our model simulates the development of an epithelial tissue from a single cell over a specified number of division cycles according to these assumptions.

### Modeling the division order

In each batch of simulations, one of two different assumptions about the order of cell division within each division cycle is made:

1. ‘Random’—The order is chosen uniformly at random from all possible orderings.
2. ‘Strict’—If a cell divides earlier in a division cycle, its daughters will also divide earlier in the next division cycle. Specifically, consider any two cells *a* and *b* that will divide in the same division cycle. If cell *a* divides earlier than cell *b*, then in the next division cycle, the daughters of cell *a* will also divide earlier than the daughters of cell *b*, and the division order between one cell’s two daughters in the next cycle is uniformly random.

The former assumption had been made throughout [3], while the latter is a new one. More detailed models of development often consider size-dependent division rates. These cannot be directly incorporated into a topological model where cell size is not a variable. However, larger size would be positively correlated with an earlier division time during the previous cycle. The ‘Strict’ division order makes the correlation between the times of consecutive divisions of each cell as large as is possible for a model with distinct division cycles.

### Modeling the choice of a cleavage plane

**Fig 1:**
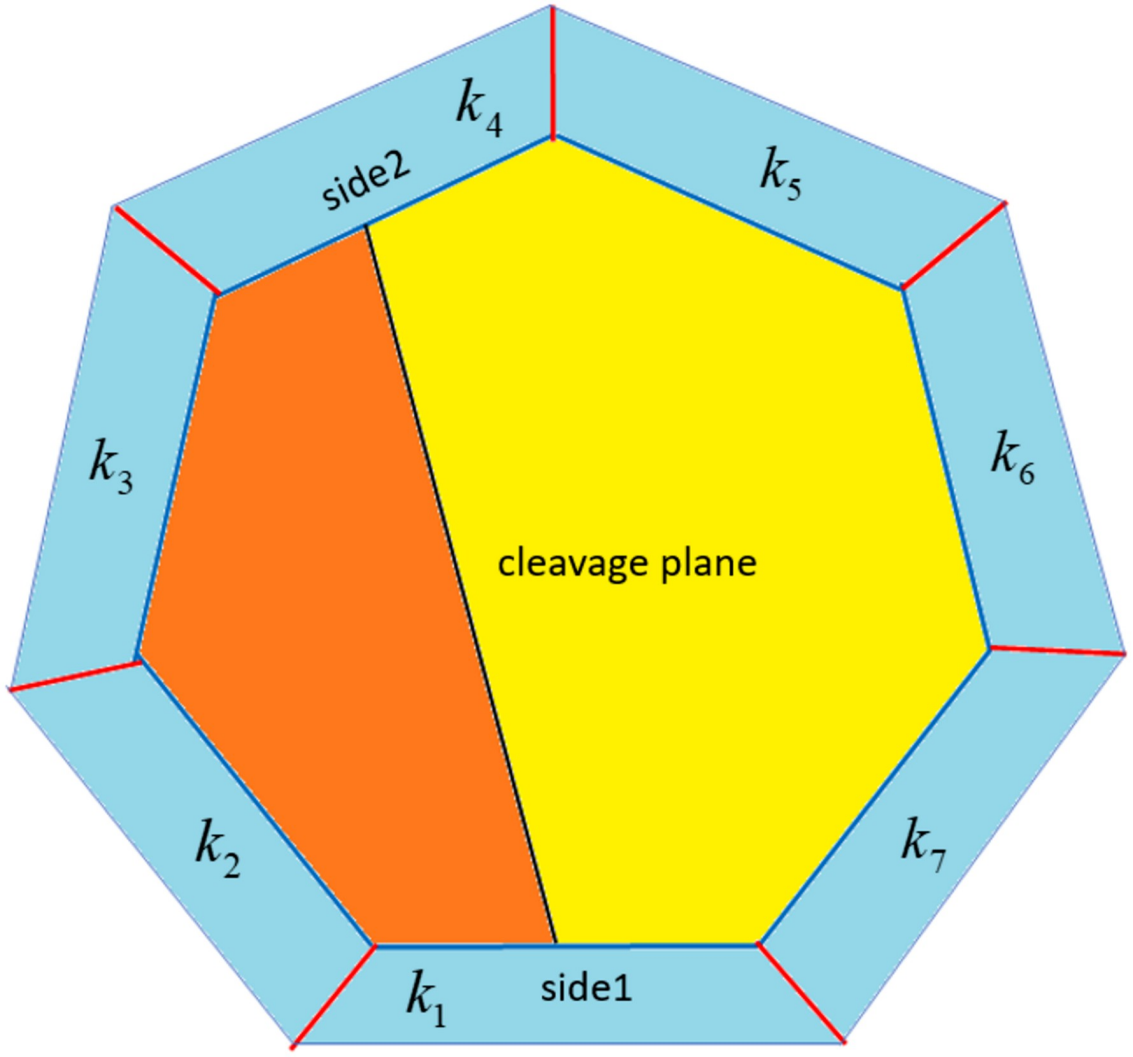
The cleavage plane is determined by choosing side1 and side2.

**Fig 2:**
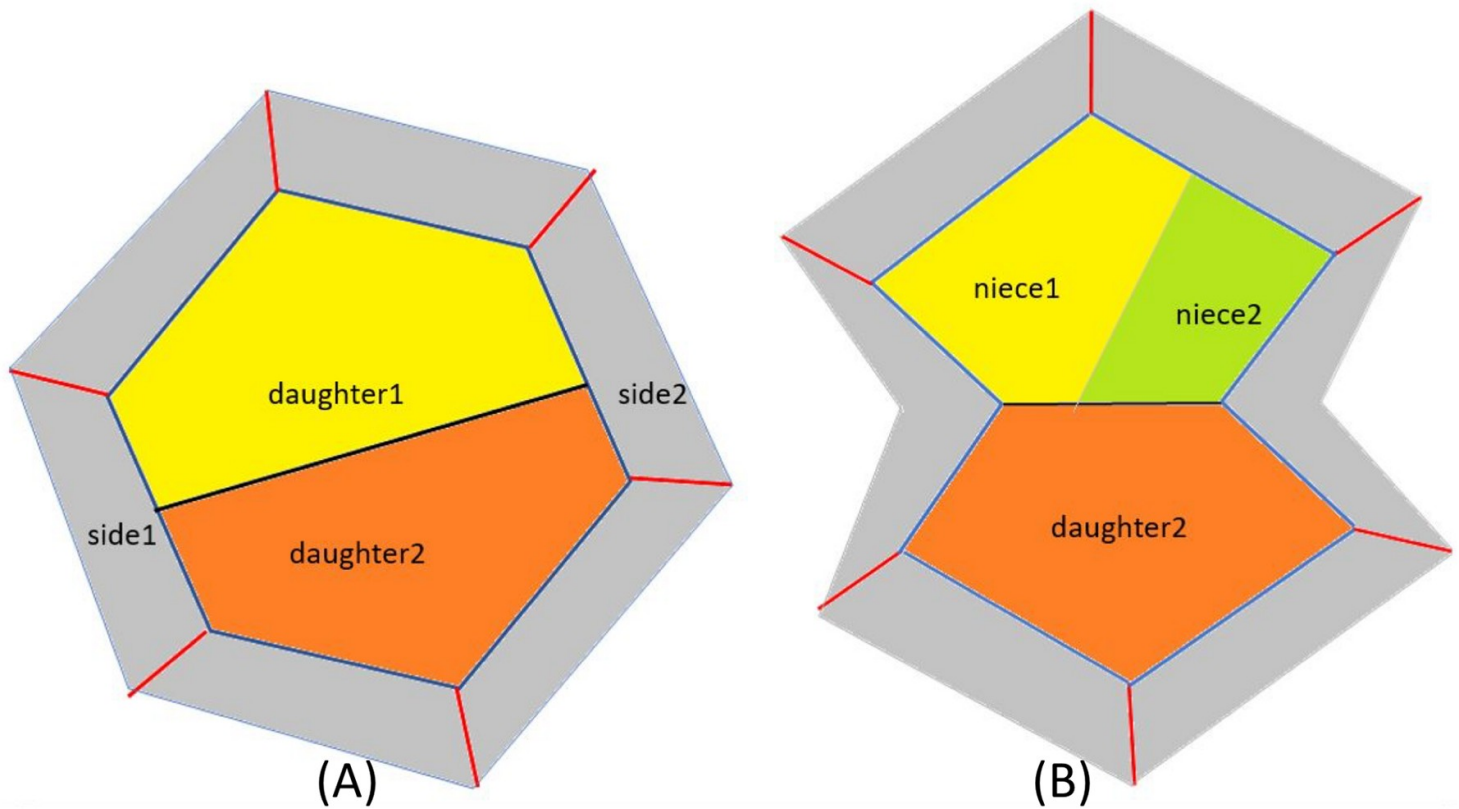
An example of two consecutive cell divisions.

To model the division of a single cell, the key is the choice of the cleavage plane, which boils down to the choices of the two edges containing the endpoints of the line segment representing it in two dimensions. For each edge of a cell, if there is a neighbor-cell on the other side, this neighbor will be unique. Therefore, an edge of a cell can be represented by this neighbor. Fig 1 illustrates this representation, and Fig 2 illustrates the process of two consecutive divisions in terms of our model.

For ease of description and to simplify wording, *side1* and *side2* will variously refer either to the two neighboring cells at which the cell will be divided, or to the edges that the cell shares with these neighbors. Side1 is chosen first according to a strategy coded by a character string ‘Choice1’ in our software, and then side2 is chosen according to a strategy coded by a character string ‘Choice2’.

#### Options for ‘Choice1’—Choosing side1

Let m denote the cell under division.

The following options were already considered in [3]:

1 ‘LaN’—Side1 is chosen to be the neighbor cell of m with the most edges. If there are several such neighbors, then choose one of them randomly.
2 ‘SmN’—Side1 is chosen to be the neighbor cell of m with the least number of edges. If there are several such neighbors, then choose one of them randomly.
3 ‘RandN’—Side1 is chosen uniformly randomly among m’s neighbors.

In [3] also an option ‘ORTHOGONAL1’ for choosing side1 was considered. The description given in [3] specifies that under this option side1 is chosen to be the edge that m shares with its sister cell. However, it remains unclear how the choice in this option is made for the sister cell that divides later and shares one such edge with each of its nieces (see Fig 2B). In order to resolve this ambiguity, we distinguished between the following more specific options for ‘Choice1’ that all conform to the description of ‘ORTHOGONAL1’ in [3]. The precise meaning of these options is best understood in the visual context of Fig 2.

4 ‘OrthSmN’—Side1 is chosen to be the edge that m shares with its sister cell. Between two sister cells, for the one that is divided later, we choose the edge it shares with the niece who has fewer edges than the other (in our algorithm, if its two nieces have the same number of edges, then choose the one who retained the label of their mother).
5 ‘OrthLaN’—Side1 is chosen to be the edge that m shares with its sister cell. Between two sister cells, for the one that is divided later, we choose the edge it shares with the niece who has more edges than the other (in our algorithm, if its two nieces have the same number of edges, then choose the one whose label is different from their mother’s).
6 ‘OrthRandN’—Side1 is chosen to be the edge that m shares with its sister cell. Between two sister cells, for the one that is divided later we choose randomly between the nieces with probability 0.5.
7 ‘OrthBornSmN’—Side1 is chosen to be the edge that m shares with its sister cell. Between two sister cells, for the one that is divided later, we choose the edge it shares with the niece who had fewer edges than the other when the nieces were born (if its two nieces have the same number of edges, then choose the one who retained the label of their mother).
8 ‘OrthBornLaN’—Side1 is chosen to be the edge that m shares with its sister cell. Between two sister cells, for the one that is divided later, we choose the edge it shares with the niece who had more edges than the other when the nieces were born (if its two nieces have the same number of edges, then choose the one whose label is different from their mother’s).
9 ‘OrthSmpN’—Side1 is chosen to be the edge that m shares with its sister cell. Between two sister cells, for the one that is divided later, we choose the edge it shares with the niece who has fewer edges than the other with probability smp (if its two nieces have the same number of sides, then choose the one who keeps the label of their mother with probability smp).

#### Options for ‘Choice2’—Choosing side2

The following options were already explored in [3]:

1. ‘evensplit’—In this option the cells will be divided as evenly as possible.
2. ‘random’—In this option, side2 is chosen uniformly randomly among the edges that are not adjacent to side1.
3. ‘unevensplit’—In this option the cells will be divided as unevenly as possible, under the restriction that an edge adjacent to side1 cannot be selected.
4. ‘Binomial’—In this option, side2 is chosen according to a binomial distribution from all edges not adjacent to side1. More precisely, the probability that side2 is chosen as the *u*^*th*^ edge from side1 in counterclockwise direction is equal to the probability of *u* − 2 successes in *i* − 4 independent trials with success probability 0.5 in each trial, where *i* is the number of sides of the cell that is to be divided.

In the option ‘evensplit’ the cleavage plane is chosen so that it divides the cell in half as closely as possible. Empirical and modeling studies indicate that this is the default, but that on occasion a different orientations would occur. The option ‘Binomial’ models such random deviations from the default. We also explored the following new options for ‘Choice2’ that correspond to this phenomenon:

1. ‘Even-Binomial’—In this option, with probability 1—probB, side2 is chosen so that a cell is divided as evenly as possible; with probability probB, side2 is chosen according to a binomial distribution from all edges not adjacent to side1.
2. ‘rotTanNorm’—Here we assume that the cleavage plane will divide the cell perfectly evenly if there is no perturbation; and if there is, the plane will rotate about the midpoint of side1. Let *β* denote the angle that the plane is rotated by. Here we assume that the probability distribution of tan(*β*) is normal, where the mean is always 0 and the standard deviation is the user-definable parameter stdbeta.
3. ‘rotNorm’—Here the same assumptions are made as in ‘rotTanNorm’ with only one exception: instead of tan(*β*), in this option, *β* is normally distributed.

**Fig 3:**
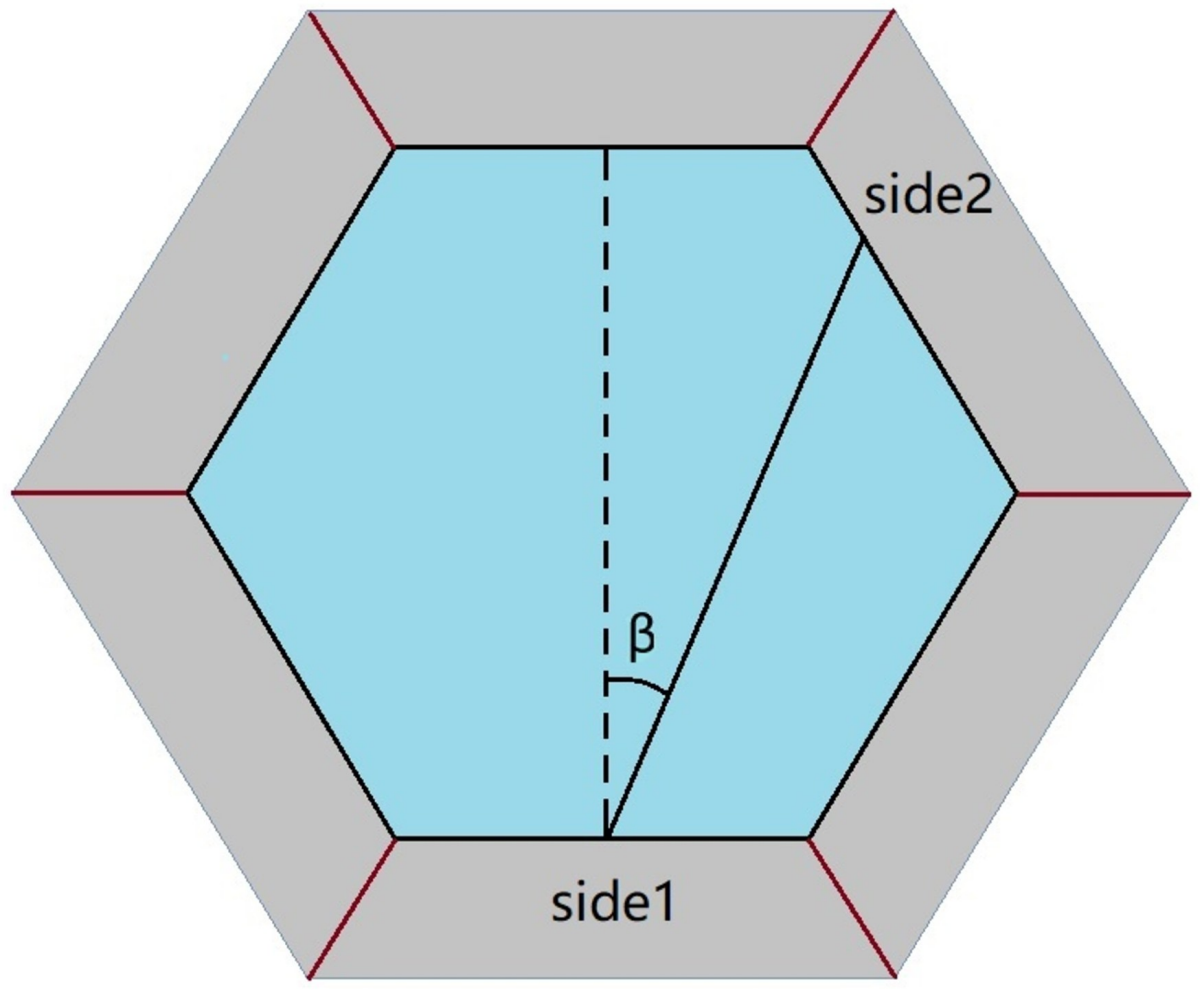
The meaning of the angle *β*.

**Fig 4:**
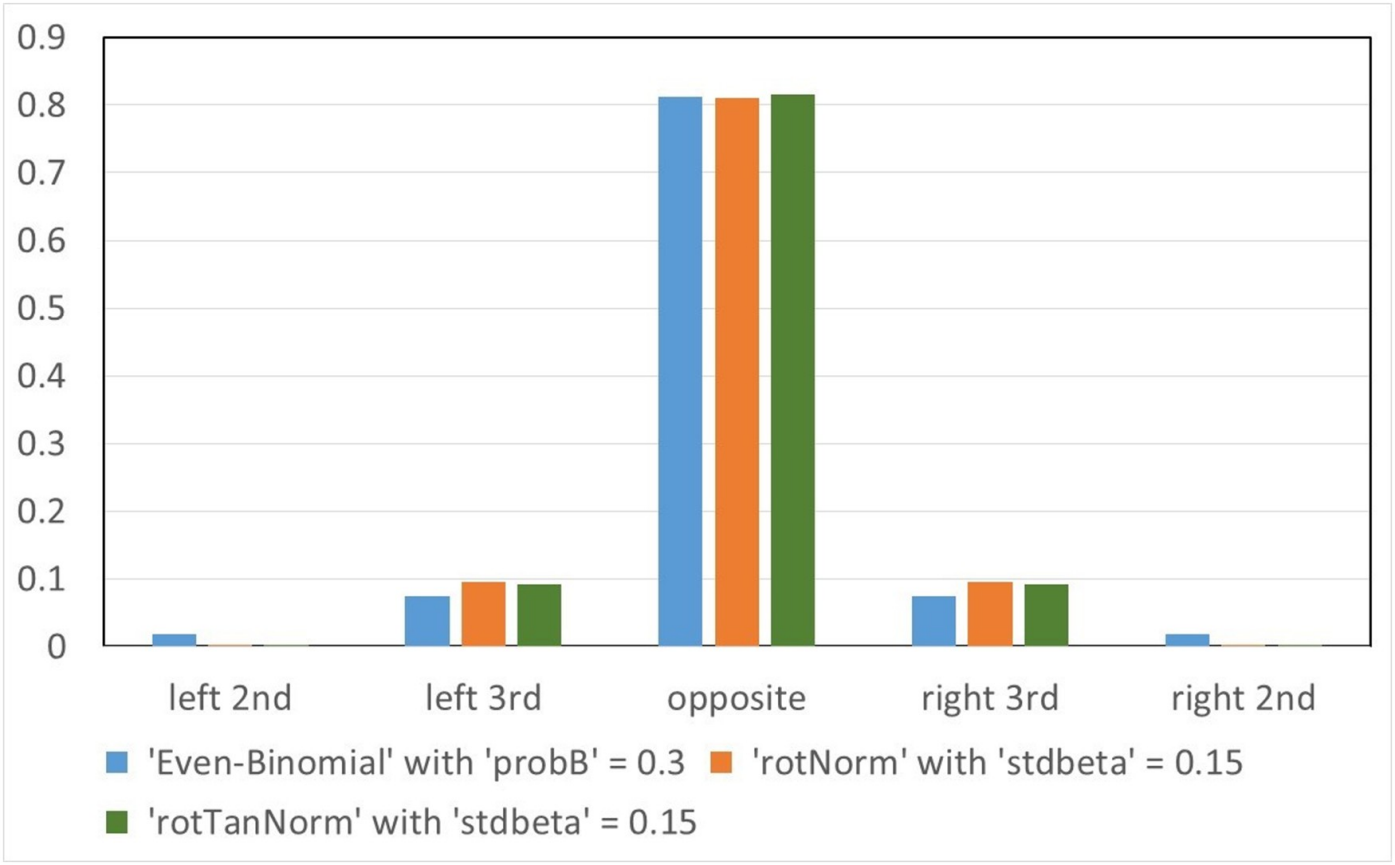
Sample probability distributions of the choice of side2 for ‘Choice2’ options ‘Even-Binomial’, ‘rotNorm’ and ‘rotTanNorm’. Relevant parameters (for an 8-sided cell): ‘probB’ = 0.3 for ‘Even-Binomial’, ‘stdbeta’ = 0.15 for ‘rotNorm’, and ‘stdbeta’ = 0.15 for ‘rotTanNorm’.

The option ‘Even-Binomial’ was already described in the supplementary material of [3], but in our opinion it is difficult to justify biologically. The options ‘rotTanNorm’ and ‘rotNorm’ were designed by us in an attempt to incorporate better approximations to empirically confirmed cleavage patterns into our modeling framework. The Discussion section gives more information on biological relevance. The meaning of the angle *β* in these options is illustrated in Fig 3. Fig 4 and the spreadsheet in S2 File illustrate the differences in the probability distributions for these options.

#### Our simulation algorithm

Here we give a brief description of the algorithm, coded in MatLab and called CellSides, that we used for simulating tissue development. A complete documentation can be found in S1 Appendix of the supplementary materials. The files of the source code are also included in the S1 File.

Each run of this algorithm simulates Batchnumber *experiments* of epithelia tissue growth from a specified initial state for Cycle division cycles. Here Batchnumber and Cycle are parameters that the user can choose via a GUI (graphical user interface) at the beginning of each run, together with the initial state, options for choosing side1 and side2, the division order (‘Strict’ or ‘Random’), and parameters smp, stdbeta, probB.

At each step of each experiment, the state of the system is represented by a vector S so that S(i) represents the number of neighbors of cell number i. Cells with S(i) = 0 are *fake cells* that represent outer edges of the growing tissue; cells with S(i) > 0 will be called *real cells*. For example, if Fig 2 represents the start of a simulation that begins with a single six-sided cell, then the grey-colored outer regions represent fake cells, while real cells are filled with the more vivid colors. The neighborhood relation between cells is represented by a matrix N. When the cells labeled *i* and *j* are neighbors, N(i,j) = 1; otherwise, N(i,j) = 0. The division order is specified by a vector O that represents a permutation of the real cells after the previous cycle. Note that the fake cells will never be divided, therefore they are not included in the vector O.

At the beginning of each experiment, the algorithm initializes these variables so as to create the initial state that has been specified by the user.

The algorithm then simulates Cycle simulation cycles. For each division cycle, the algorithm performs the following updating steps:

- First it updates O. When the chosen division order is ‘Random’, it uses a random permutation of all real cells that are present at the beginning of the current cycle. When the chosen division order is ‘Strict’, it uses independent random permutations of each pair of sisters, where each pair of sisters is divided consecutively, and cells from different pairs are divided in the same order as their mothers in the preceding cycle.
- Then it divides each cell in the order specified by O. To divide cell m, it needs to successively:
  1. Extract relevant information about the neighbors of m from S and N.
  2. Choose side1 according to the option chosen by the user.
  3. Choose side2 according to the option chosen by the user.
  4. Update S for the daughters of m and the cells across the edges that represent side1 and side2.
  5. Update N.

At the end of cell division cycles number 9, 10, and 11, when Cycle is greater than 9, 10 or 11 respectively, as well as the end of all the division cycles, the algorithm will collect the data we are interested in. Finally, these data will be saved to output files.

### Data collection and data analysis

The data for each run of CellSides are saved in subfolders of a folder named ‘Data’. The names of these subfolders identify the relevant input parameters.

To extract and analyze the parts of these data that are discussed here, we wrote two MatLab scripts described below. These scripts can also be used for ranking the outcomes of our simulations in terms of fit with empirical data sets other than those used as our benchmarks. For detailed instructions on how to use these scripts see the source code in the S1 File.

- RunChi2Alltogether.m—This script calculates two *χ*^2^ statistics that compare the polygonal distribution of the simulation data in our simulations to each of the polygonal distribution of the empirical data for *Drosophila, Hydra, Xenopus*, Cucumber and *Anagallis*, and creates a file named Chi2.xls for each of them in the corresponding folders. Only the distributions of *k*-sided cells for 4 ≤ *k* ≤ 9 are taken into account in this calculation.
  The file has two sheets. In Sheet 1, the denominators used for calculating the *χ*^2^-statistics are based on empirical data; in Sheet 2, the denominators used for calculating the *χ*^2^-statistics are based on the simulation data.
- rankfolders.m—Ranks the folders containing our simulation data according to the *χ*^2^ statistics stored in Chi2.xlsx for any of the five species from the smallest to the largest. The rankings are based on Sheet 2 of the files Chi2.xls in order to avoid some artifacts that may result from very low frequencies of *k*-sided cells for some relevant *k* in the empirical data. The ranking is done separately for each of the five species, with the species being specified by the user for each run of the code.

### Parameter choices for our simulations

We ran and analyzed a total of 1076 batches of 100 experiments each. It had already been reported for the model of [3] that the final distributions are fairly insensitive to the number of sides of the initial cell, and preliminary explorations of our model had confirmed this. Therefore in each of these 1076 batches, the initial state was set to a single 7-sided cell.

We run simulations for all pairings of options ‘SmN’, ‘OrthSmN’, ‘OrthLaN’, ‘OrthRandN’, ‘OrthBornSmN’, ‘OrthBornLaN’ for choosing side1 with options ‘evensplit’, ‘random’, ‘Binomial’, ‘rotTanNorm’, ‘rotNorm’ for choosing side2, both under the ‘Random’ and ‘Strict’ division orders. As ‘OrthSmpN’ was conceived as an interpolation between ‘OrthSmN’ and ‘OrthRandN’ that might further improve the best fit, we paired it only with the options ‘evensplit’, ‘rotTanNorm’, ‘rotNorm’ that had already given better-fitting distributions than ‘random’ and ‘Binomial’, and then with ‘Even-Binomial’ for comparison purposes. For options that involve a parameter ‘smp’ or ‘stdbeta’, the range of values for these parameters was chosen dynamically so as to give a good representation of the resulting polygonal distributions.

For the sake of reproducing findings of [3], we also included some simulations with the options ‘LaN’ and ‘RandN’ that were found in [3] to give worse fits. We did not include in our 1076 batches simulations with the biologically implausible option ‘unevensplit’ that was shown in [3] to also give a poor fit. Moreover, for comparison purposes we did some simulations with the option ‘Even-Binomial’ for choosing side2, paired with our best-performing options for choosing side1. Here both the ‘Random’ and ‘Strict’ division orders were used in the interval for the parameter ‘probB’ where [3] had reported the best fit, and only the better-performing ‘Random’ order was explored for values of ‘probB’ outside of this interval.

Table in S1 Table of the supplementary material gives a complete list of all parameter settings that were explored.

## Results

The paper [3] had reported a remarkably close fit between predictions of a topological division-only model of epithelial development with empirical data on the polygonal distributions in five evolutionarily distant organisms: *Drosophila*, *Hydra*, *Xenopus*, Cucumber, and *Anagallis*. We were interested in investigating whether certain modifications of their model would give a better fit. Our first modification consisted of refining the ambiguous option ‘ORTHOGONAL1’ of [3] for choosing side1 into six distinct unambiguous ones as described above. The second modification was adding two new options ‘rotTanNorm’ and ‘rotNorm’ for choosing side2. The third modification consisted of including a new option ‘Strict’ for the division order.

We independently coded a version of the model in [3] and added these new options. We ran and analyzed a total of 1076 batches of 100 experiments each for different parameter settings as described in the preceding subsection. Each experiment was run for 12 simulated division cycles. We then took the five empirical data sets used in [3] as our benchmarks and ranked the options and parameter settings according to the *χ*^2^ statistic for best fit with the empirical data, separately for each of the five organisms mentioned above.

We confined our statistical data analysis that is presented here to the particular data sets of [3] for several reasons. First of all, this gives us a set of benchmarks that has already been previously chosen by a third party. Second, as we will argue in the Discussion, topological models based on cell division alone appear appropriate only to early stages of development, so that a comparative analysis of our options on data sets from later developmental stages where the assumptions of our model are violated would be meaningless. Third, we think of the data set of simulations that we produced as a research tool. It is being made publicly available at https://doi.org/10.5061/dryad.57n3b70 that can be used by other researchers to rank these outcomes for best fit with additional data sets of interest, including ones that may become available in the future.

Our simulations and statistical analysis of our benchmarks replicated the results in [3] reasonably well, but not entirely for option ‘ORTHOGONAL1’, which appears to have been implemented in a similar way to our option ‘OrthRandN’.

**Fig 5:**
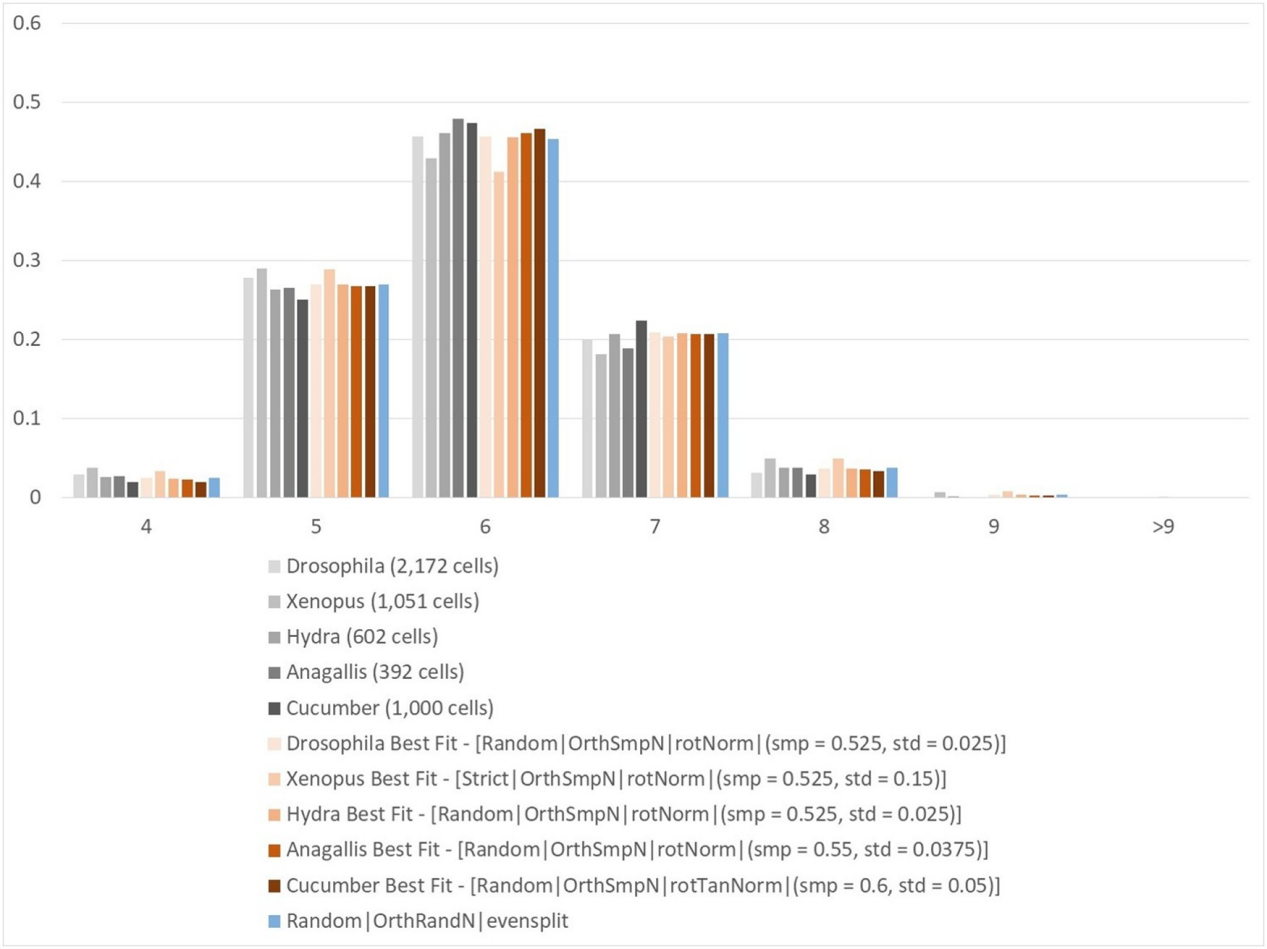
Simulated and empirically verified polygonal distributions. Gray scale: real organisms. Reddish: new strategies. Blue: best-fitting option from [3].

The histogram of Fig 5 shows the polygonal distributions for the five organisms mentioned above, together with the distributions that we get from our top scoring choices, and from the option conceptually consistent with the top-scoring one of [3]. Table 1 shows *χ*^2^-statistics for the fit of our top scoring options and of the top scoring choices that are consistent with options implemented in [3] with the five organisms. Here the options that were ranked top 3 for each organism are included.

**Table 1:** Top-ranking options.

Organism	Strategies and Parameter settings	*χ*^2^ Statistic
*Drosophila*	Random-OrthSmpN-rotNorm-0.525-0.025	0.003825
	Random-OrthRandN-rotNorm-*-0.05	0.003898
	Random-OrthRandN-rotTanNorm-*-0.05	0.003903
	**Random-OrthRandN-evensplit**	**0.003996**
	**Random-OrthSmpN-Even-Binomial-0.6-0.05**	**0.004320**
*Hydra*	Random-OrthSmpN-rotNorm-0.525-0.025	0.001602
	Random-OrthSmpN-rotNorm-0.525-0.05	0.001635
	Random-OrthSmpN-evensplit-0.525-*	0.001653
	**Random-OrthRandN-evensplit**	**0.001731**
	**Random-OrthSmpN-Even-Binomial-0.6-0.05**	**0.002157**
*Xenopus*	Strict-OrthSmpN-rotNorm-0.525-0.15	0.00363
	Random-OrthSmpN-rotNorm-0.525-0.15	0.003658
	Random-OrthSmpN-rotNorm-0.7-0.2	0.003706
	**Strict-OrthLaN-evensplit**	**0.01434**
	**Random-OrthSmpN-Even-Binomial-0.6-0.15**	**0.003816**
Cucumber	Random-OrthSmpN-rotTanNorm-0.6-0.05	0.004508
	Random-OrthSmpN-rotNorm-0.6-0.0375	0.004580
	Random-OrthSmpN-rotTanNorm-0.65-0.0125	0.004632
	**Random-OrthRandN-evensplit**	**0.008767**
	**Random-OrthSmpN-Even-Binomial-0.6-0.05**	**0.009170**
*Anagallis*	Random-OrthSmpN-rotNorm-0.55-0.0375	0.007145
	Random-OrthSmpN-rotTanNorm-0.55-0.0625	0.007310
	Random-OrthSmpN-rotNorm-0.525-0.05	0.00747
	**Random-OrthRandN-evensplit**	**0.007668**
	**Random-OrthSmpN-Even-Binomial-0.6-0.05**	**0.007709**

Top-ranking options from our simulations (lightface) and top-ranking options considered in [3] (boldface), together with *χ*^2^-statistics. When the options for choosing the cleavage plane involve numerical parameters, they are listed so that the numerical parameter relevant for choosing side1 appears first, followed by the numerical parameter relevant for choosing side2. A lower *χ*^2^-statistic signifies a better fit with the data.

These results clearly indicate that option OrthSmpN for choosing side1 with parameter smp close to 0.55, when combined with our new options rotNorm and rotTanNorm for choosing side2, consistently give significant improvements of the fit relative to the options of [3]. In contrast, with one notable exception for the data on *Xenopus*, our new option ‘Strict’ for the order of cell division tended to perform worse than the option ‘Random’ that was already explored in [3].

## Discussion

Empirically studied polygonal distributions in epithelia show remarkable similarities in a variety of evolutionarily distant organisms. This is exemplified by the organisms studied here and in [2, 3], as well as by tissues from other organisms, like *Arabidopsis* leafs [11] and early developmental stages of the chick embryo [9]. While markedly different distributions have been found in other epithelial tissues of some other species, most notably from the plants *Anacharis, Volvox* [7] and from later developmental stages of the chick embryo [9], the strong conservation of the standard polygonal distribution is a puzzling phenomenon. A variety of mathematical models that can reproduce this distribution have been proposed. Our refinement of the model of [3] is purely topological, which means that it takes into account only the number of sides of a given cell and the neighborhood relation. It also assumes that cell division is the only process by which developing tissues are modified. In contrast, geometric models such as in [16–19] and the ones reviewed in Section 8 of [1]) take into account also geometry (cell shape and/or cell size) and/or mechanical stresses [15, 20–24]. Development is a complex process and such factors as mechanical stresses certainly play a role [20, 25]. It is not immediately clear though when these processes are actually needed for explaining an empirically observed distribution. In our opinion, this would be the case only if simpler and more parsimonious models like ours give a significantly worse fit with a particular data set.

Moreover, it may not be particularly meaningful to compare predictions of division-only models as ours with models like [14, 15, 21] that include other processes, such as cell rearrangements, by which epithelial tissues can be modified. Epithelial development proceeds in distinct stages. To quote from [13]: “… the stratification of the mammalian epidermis consists of two phases: a proliferative, amplification phase in which symmetric divisions increase the surface area of the epithelium, followed by an asymmetric division phase generating distinct molecular identities …” We found indications in [2, 14, 15] that cellular rearrangements may be (largely) confined to this second phase. Thus it appears to us that division-only models may apply to tissues in earlier stages of development, while models that take incorporate rearrangements would be more biologically realistic for tissues sampled from later stages.

We therefore believe that topological division-only models like the one studied here should be viewed as a highly parsimonious default explanation of the polygonal distributions at early stages of epithelial development. Our guiding question was whether and to what extent the fit of the previously published model [3] can be improved by including more options within the same modeling framework. In particular, we were interested in exploring new options for the choice of side2. In [3], the option ‘evensplit’ was found to give the best fit. In this option, the cleavage plane is chosen so that it divides the cell in half as closely as possible. Empirical and modeling studies give support to the assumption that this might be the case most of the time, but that on occasion a different orientations would occur, where the cleavage plane cuts at an angle that is close to, but not identical to what would be predicted by ‘evensplit’ [26–30]. In particular, [26, 28] support distributions that would correspond to rotation by a small random angle of the mitotic spindle that is anchored near the center of mass of the cell. Our options ‘rotNorm’ and ‘rotTanNorm’ work in exactly this way. Mathematically they are equivalent to randomly perturbing the center of the spindle to a position that would be the midpoint of the resulting cleavage plane, and then rotating the default orientation of the unperturbed spindle by an angle *β*. It might seem more natural to consider instead distributions where the perturbation of the spindle center from the center of mass of the cell is independent from the perturbation of the angle. But this would require introduction of at least one additional parameter and a considerable amount of guesswork, since in the context of topological models there is no clear-cut notion of the center of mass of a cell. Thus it seemed prudent to work with admittedly imperfect distributions that have natural interpretations within our framework and keep the number of parameters as small as possible so as to avoid overfitting.

Similar deviations had previously been modeled in [2, 3, 9] by assuming a binomial distribution or an interpolation ‘Even-Binomial’ between the binomial distribution and ‘evensplit’. However, the usual justification for a binomial distribution, that the number of edges between side1 and side2 would be determined by randomly and independently drawing such edges from the feasible set, does not make biological sense. To see why, consider Fig 1. Here we would draw from the set {*k*_3_, *k*_4_, *k*_5_, *k*_6_} and our cleavage plane would result from two “successes.” A biologically meaningful situation would come from drawing {*k*_3_, *k*_4_}, but not from drawing {*k*_3_, *k*_5_} or {*k*_3_, *k*_6_}.

It is interesting to compare our options ‘rotNorm’ and ‘rotTanNorm’ with one another and with ‘Even-Binomial’, which was proposed in [3] as a promising alternative to ‘evensplit’, but consistently gives worse fits (see Table 1). Fig 4 gives an illustration: ‘rotTanNorm’ and ‘rotNorm’ tend to be very similar to each other and more strongly peaked at the mode than ‘Even-Binomial’. The spreadsheet in S2 File of the supplementary material gives a comprehensive comparison between these distributions.

As Table 1 shows, our new options ‘rotNorm’ and ‘rotTanNorm’ for choosing side2 did significantly outperform previously described options on the data sets that we took as our benchmarks. In view of the above theoretical considerations that led us to their design, this was expected.

The finding that for choosing side1 always option OrthSmpN with parameter smp near 0.55 worked best came as a bit of a surprise for us. We had designed this option simply as one of six unambiguous implementations of the option ‘ORTHOGONAL1’ of [3], and had not expected this level of consistency of the outcomes. In this option, when the two nieces do not have the same number of sides, then the new cleavage plane cuts a side of the niece with fewer edges with probability 0.55, just a little more than half of the time. This seems somewhat plausible, since the number of sides of a cell would be negatively, albeit perhaps weakly, correlated with the length of the common edges that these nieces share with the focal cell.

The ‘Random’ division order of [3] may be appropriate when cell division timing is tightly synchronized, as has been reported for early *Drosophila* embryonic development in [31], and totally independent of previous division times. On the other hand, cell-size dependent division rates in epithelial tissues have been empirically confirmed [10, 32] and incorporated into models [10, 16], even to the point where the notion of temporally separated division cycles no longer applies. In contrast to most geometrical models, cell size does not directly enter topological ones. However, it seems plausible to assume that cells whose most recent division occurred earlier tend to be larger. One can think of our ‘Strict’ division order as enforcing the the largest correlation between consecutive division times of a given cell that is possible when cells divide in distinct cycles. We were surprised that it usually performed worse than ‘Random’. Quite possibly, it assumes too strong a correlation, and an appropriate interpolation between the ‘Random’ and ‘Strict’ division orders would be biologically more realistic.

In summary, we found that the fit of the model of [3] with their empirical data can be substantially improved by considering additional, and presumably biologically more realistic, options for choosing side1 and side2. These modifications perform better than previously studied ones and lead to remarkably close fits with the distributions from our set of benchmarks. We want to emphasize that our statistical analysis serves only the purpose of benchmarking and is not to be taken as a comprehensive analysis of all potentially relevant empirical data sets. Analysis of additional empirical data sets for fit with our simulation data, including ones that may become available in the future, can be performed independently. The data set of simulations that we produced is being made publicly available at https://doi.org/10.5061/dryad.57n3b70 and the scripts for data analysis are included with the source code in the supplementary materials S1 File.

It may also be possible in future work to further refine our new options to obtain an even better fit within the confines of topological division-only models.

## Conclusion

A variety of mathematical models have been proposed for explaining the polygonal distribution in developing epithelia that is fairly strongly conserved across evolutionarily distant species. Our refinement of the model of [3] is purely topological, which means that it takes into account only the number of sides of a given cell and the neighborhood relation between cells. It also assumes that cell division is the only process by which developing epithelial tissues are modified. Development is a complicated process and many factors play a role. A number of more elaborate models have been proposed that incorporate cell geometry (shape and size), mechanical stresses, and/or processes like cell arrangements. However, for a phenomenon that is so strongly conserved across distant taxa, one would naturally be interested in how much of it can be explained by the most parsimonious kind of model, like the topological models studied here. To quote [33] on [2]: “The steady-state polygon distribution can therefore be regarded as an emergent property of the process by which cells replicate; the result requires no consideration of surface free energy and only assumes that cell adhesion is stable through cell divisions.”

Our work shows that by including certain more biologically plausible options into the model of [3], the goodness of fit with empirical distributions can be substantially improved without leaving the parsimonious framework of topological models based on division only. This framework may be appropriate for early developmental stages of epithelia. Moreover, it can serve as a default explanation with which the predictions of more detailed models can be compared. Our algorithm and simulation data can be used as tools in such comparative studies.

## Supporting information

S1 Appendix A complete description of the software used in our simulations. (PDF)

S2 Appendix A technical result that is needed for the proof of correctness of our code. (PDF)

S1 File Source code. (ZIP)

S2 File Spreadsheet with comprehensive comparison of distributions for choosing side2 under options ‘rotTanNorm’, ‘rotNorm’, and ‘Even-Binomial’ for a wide range of choices of the parameters stdbeta and probB. (XLSX)

S1 Table A complete list of all parameter settings that were explored. (PDF)
