# Supplementary material for: Development of epithelial tissues: How are cleavage planes chosen?
Source: PLoS One. 2018 Nov 7;13(11):e0205834. doi: 10.1371/journal.pone.0205834 (PMC6221281; doi:10.1371/journal.pone.0205834)
Supplement: S1 Appendix — (PDF) [file pone.0205834.s001.pdf]

Simulating the process of cell division with MatLab: Documentation of the software

Winfried Just and Ying Xin
Department of Mathematics, Ohio University

The package `cell division` consists of script m-files that simulate the process of monolayer cell division and perform some analyzes of the data.

In order to make our programs easier to understand, there are more lines of code than strictly necessary and ample comments in the m-files themselves.

1 Files in the package `cell division`

1. `CellSides.m` — This is the master script. First it calls `SetParam` which allows users to choose parameters via a dialogue box. Hence the division order, the initial state S, the initial neighborhood matrix N, the method of choosing side1 and the method of choosing side2 which is represented by the probability distribution of I denoted by P, the number of division cycles in each experiment, the standard deviation of the normal distribution, the batch size, and the probabilities `smp` and `probB` are set according to the specified parameters. Then the script will call `AnyOrderCellSides.m`, which is the main file that will execute the simulations.

2. `SetParam.m` — Lets user choose parameters for the model via a GUI. It allows user to choose the values of some parameters via an interface, including the division order, the initial state `S`, its corresponding neighborhood matrix `N`, the methods of choosing side1 and side2, the number of cell division cycles in each experiment, the standard deviation of the normal distribution when a normal distribution is needed, the batch size, `smp` and `probB`.

3. `AnyOrderCellSides.m` — This is the main file that will execute the simulations.

4. `DataCollection.m` — This file is called by `AnyOrderCellSides.m` when a specified number of cell division cycles in an experiment have been completed. It computes a number of statistics on the current state of the tissue and saves them to corresponding output variables.

5. `DataReport.m` — Called by `AnyOrderCellSides.m` at the end of each batch of simulations. Saves the data of interest and displays some of them.

6. `RunChi2Alltogether.m` — After all the simulations are done, the script calculates the χ^2 statistic with the polygonal distribution of the simulation data (in our simulations,

this is the vector `MEANDistribution4to9 and larger`) compared to each of the polygonal distribution of the empirical data for *Drosophila, Hydra, Xenopus,* Cucumber and *Anagallis,* and creates a file named `Chi2.xls` for each of them in the corresponding folders.

7. `rankfolders.m` — Ranks the folders containing our simulation data according to the χ^2 statistics stored in `Chi2.xlsx` for any of the five species from the smallest to the largest.

2 Syntax

- To run a batch of simulations:

  1. Enter in the Command Window:
     `>>  CellSides.m`
  2. Set parameters in the dialogue box according to the available options displayed in the Command Window so that the division order, the initial state `S`, its corresponding initial neighborhood matrix `N`, the strategies of choosing side1 and side2, the number of division cycles, the standard deviation of the normal distribution (when a normal distribution is needed), the batch size and the values of `smp` and `probB` that one would like to explore are defined.
  3. Click **OK**.

- For the calculation of the χ^2 statistic, save `RunChi2Alltogether.m` in the parent directory of the `Data` folder and enter in the Command Window:
  `>>   RunChi2Alltogether.m`

- To rank the resulting folders according to the χ^2 statistic for a particular organism:

  1. Save `rankfolders.m` in the parent directory of the `Data` folder.
  2. Modify lines 41 and 49 of the script so that it works for the given species; see comments in the source code for details.
  3. Enter in the Command Window:
     `>>   rankfolders.m`

3 Detailed description of individual scripts and functions

Here we focus on the code that does the actual simulations. The code for data analysis is straightforward, with comments in the m-files themselves providing explanations when appropriate.

1. `AnyOrderCellSides.m` — This is the main file that will execute the simulations.

   The following functions that are defined and called inside this script:

   - `initialS*` — Defines the initial state using a row vector. Here $* \in \{4, 5, ..\}$ stands for the number of sides of the unique initial cell. For each cell, we need to know two

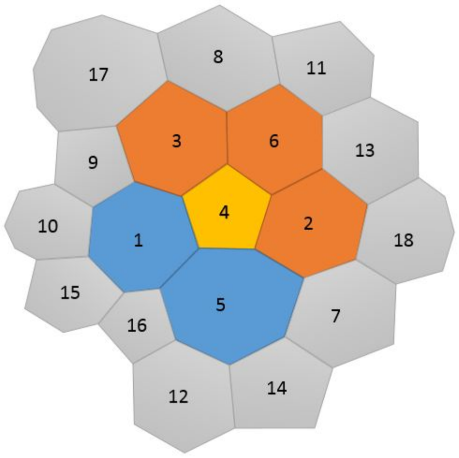

things about it: its label and the number of its edges. In the initial state `S`, the ith element represents the cell labeled i and the value of this element is the number of edges of the cell. Further, we want to use the neighbors of a cell to represent the edges, but for some edges of some cells (the outermost ones), there is no neighbor on the other side. To solve this problem, we introduce the concept of a "fake cell." That is, for edges without neighbor on the other side, we pretend that there were neighbor cells, and the number of edges of these imaginary cells is always zero. For example, suppose a simulation is started with a single cell that has 6 edges. Then we label this cell as cell number 1. It has 6 edges, no neighbor, so we will need 6 fake cells labeled 2, 3, 4, 5, 6, 7. Therefore, the initial state will be defined as the row vector $[6, 0, 0, 0, 0, 0, 0]$.

- `initialN*` — Defines the initial adjacency matrix. Throughout the simulations it will be updated after each cycle for cell division. This matrix and its updated versions will be denoted by `N`, and $* \in \{4, 5, ..\}$ stands for the number of sides of the unique initial cell. The initial `N` is uniquely determined by the initial state `S`. If the cells labeled i and j are neighbors, `N(i,j)=1`; otherwise, `N(i,j)=0`.
- `initialSm` and `initialNm` — Define the initial state and the initial neighborhood matrix corresponding to the following initial condition respectively:
  The numbers in the figure are labels of the cells. Here "fake cells" are grey. These initial states and matrices were only used by us in preliminary explorations.
- `PrI` — This function computes the probability distribution of `I`, according to the option for parameter `Choice2` for choosing side2 as specified by the user. Valid choices are described in the main text.
  The variable `I` represents the number of edges between the two chosen edges at which the cell will be divided (these two edges are not counted in `I`).
  Note that if a cell is not divided totally evenly, there will be two possible such `I` values, and here `I` is always chosen as the smaller one. For example, in Figure 1, $I = 2$. The output of `PrI` is a matrix, and the ith row is the probability distribution of `I` for cells with i edges. Since one-, two- and three-sided cells are excluded by our assumptions, the first three rows are never used, but included for easier readability

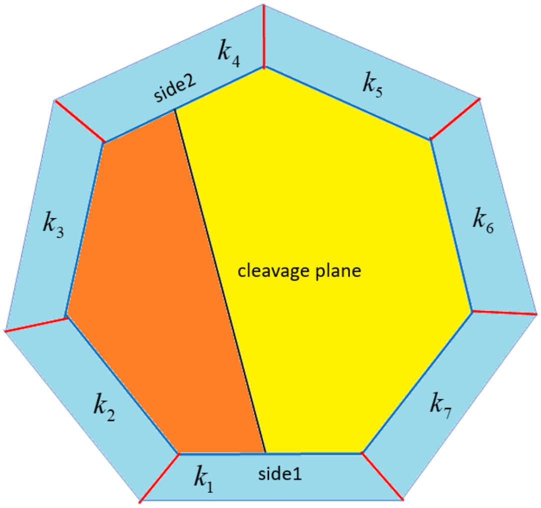

Figure 1: The cleavage plane is determined by choosing side 1 and side2.

of the code. When certain strategies of choosing side1 and side2 are applied, cells with very large number of sides are more likely to occur. In order to avoid undefined distributions, the range of i is chosen differently for different options of choosing side1 and side2.

- `probbeta.m` — For the probability distributions that use a normal distribution in `PrI`, this function file defines the *cdf* of the normal distribution.

For each call of `CellSides`, this algorithm simulates `Batchnumber` *experiments* of epithelia tissue growth from the specified initial state for `Cycle` division cycles. At each step, the state of the system is represented by a vector `S` so that `S(i)` represents the number of neighbors of cell number `i`. Cells with `S(i)` $= 0$ are *fake cells* that represent outer edges of the growing tissue; cells with `S(i)`> 0 will be called *real cells.* The neighborhood relation between cells is represented by a matrix `N`, so that the cells labeled i and j are neighbors, `N(i,j)` $= 1$; otherwise, `N(i,j)` $= 0$. The division order is specified by a vector `O` that represents a permutation of the real cells after the previous cycle. Note that the fake cells will never be divided, therefore they are not included in the vector `O`.

The initial state is chosen using the functions `initialS*` and `initialN*`.

For each division cycle, the algorithm performs the following updating steps:

- **Update `O` depending on chosen `DivisionOrder`:**
  - Under the `'Strict'` option, the division order of the first cycle among the initial cells is random. In later cycles, considering any two cells a and b, if cell a divides earlier than cell b, then in the next cycle, the daughters of cell a will also divide earlier than the daughters of cell b, and the division order between one cell's two daughters in the next cycle will be uniformly random. Therefore, after each cycle, the length of the current `O` (denoted by `w`) and the difference `l`

of the length of the updated `S` and `w` are recorded, so that we know that in the next cycle, we will need to add `w` more cells to `O`. In the newly updated `S` these are the cells labeled `l+1,l+2,...,l+w`, and their sisters are the cells labeled `O(1),O(2),...,O(w)` respectively. So when defining the new order `O` for the next cycle, for each `i`, we just need to insert the label `l+i` to the left or the right of its sister.

- Under the `'Random'` option, `O` is chosen uniformly at random from all possible orderings. However, we still need to use a similar strategy as in `'Strict'` to keep track on the sisterhood among the cells, and it is represented by a row vector `Os`. This time, unlike in `'Strict'`, Os is defined based on the order vector O of the previous cycle instead of Os itself. Here we will always insert the label `l+i` to the right of its sister label in `Os`. Therefore, for any positive integer k, as long as $2k$ is less than or equal to `2w`, cells `Os(2k-1)` and `Os(2k)` are sister cells. Then, *inside each cycle*, the order defined by `O` is applied and the cells `O(1),O(2),...,O(n)` will be divided one by one using a loop, where `n` is the number of real cells.

- **Divide each cell in the order specified by `O`:** To divide cell `m`, the algorithm needs to choose the two edges that would contain the endpoints of the cleavage plane. Again, the first edge (neighbor) chosen is denoted by side1, and the second is denoted by side2.
  - **Choose side1:**
    In order to choose *two* neighbors representing two edges of `m`, the algorithm first needs to find *all* neighbors of it. The neighborhood is implicitly defined by `N`. The algorithm extracts this information from `N` and forms a row vector `nei` that lists the labels of these neighbors in an order so that `nei(i)` and `nei(j)` will share a vertex if, and only if, $|i-j| \leq 1$ or $|i-j|+1$ is the length `S(m)` of `nei`. It also records the cells that are not `m`'s neighbors, including `m` itself, as `nonnei`. Then, based on `N` and `nei`, the algorithm gets a submatrix of `N` representing the neighborhood relationship among `m`'s neighbors and lets `M` denote this matrix. Now the algorithm uses a `switch` command to choose side1 according to the user-defined option `Choice1` for choosing side1.
  - **Choose side2:**
    Now suppose side1 is chosen. Let `j(1)` be the label so that side1 is the $j(1)^{th}$ element in `nei`. Note that it is important that we work with `nei` and `M` here instead of `S` and `N`.
    We choose side2 by first choosing `I`, the number of edges between side1 and side2. For example, in Figure 1 we have $I = 2$. Generate a random number `r` and then we can choose `I` based on the probability distribution that applies, which is the $S(m)^{th}$ row of the matrix `P` that was computed at the start of the simulation by the function `PrI`.
    Once `I` is chosen, we just need to chose one side of `j(1)` that has exactly one common vertex with `m`. Recall that we assumed that cells with three edges will not occur in the simulation. Thus by the lemma of Appendix S2, here will be exactly two such sides. This defines an orientation and a neighbor cell `j(2)`. Cell k_2 is this neighbor cell `j(2)` in Figure 1. Following this orientation, with

side1 and this first neighbor cell between side1 and side2 chosen, the algorithm recursively finds the two neighbors of the newly chosen cell among neighbor cells (cell k_1 and cell k_3 in Figure 1), where the one that is not chosen in `j` yet is the next cell we need (cell k_3 in Figure 1) until it gets the whole path which ends at side2. Once this process is complete, we get a row vector `j` whose first element is side1, the last element is side2, and all the other elements are edges sitting in between.

Again, note that `j(i)`is the ith neighbor in `nei`, not the label of the cell in `S`. Hence we still need one more step to convert the labels back into labels in `S`. The vector we get from this step is denoted by `PATH`. This vector `PATH` is a complete path that includes side1, cells between the edges to be divided, and side2. In Figure 1, we have `PATH = [`k_1, k_2, k_3, k_4`]`.

– **Update `S`:**

After the `PATH` is chosen, the cell will be divided into two daughter cells. In terms of their labels, we retain the label `m` for the daughter cell that is adjacent to the $PATH$ and label the other daughter as cell `length(S)+1`.

To update the state vector `S` and the neighborhood matrix `N`, we partition all the cells into five classes:

(a) daughter cells;

(b) `endcell` – end points of `PATH`, that is, side1 and side2 (in Figure 1, these are k_1 and k_4);

(c) `pathcell` – cells sitting between side1 and side2 (in Figure 1, these are k_2 and k_3);

(d) `npneicell` (non-path neighbor cell) – cells that were neighbors of the mother cell `m` but are not in `PATH` (in Figure 1, these are k_5, k_6 and k_7) ;

(e) `nonneicell` (non-neighbor cell) – these are all the other cells.

Now we update `S` as follows:

* The daughter cell `m` has `I+3` sides and therefore `S(m) := I+3`;
* The daughter cell `length(S)+1` has `S(m)+1-I` sides, so `S(length(S)+1) := S(m)+1-I`;
* The numbers of side1 and side2's edges are increased by 1, hence `S(endcell) := S(endcell)+1`;
* Note that a fake cell can be chosen as an `endcell`, so the fake cells may gain edges and become indistinguishable from the real cells. To keep the fake cells "fake," suppose a fake cell was chosen to be an `endcell`. Then it will gain one more edge and hence have no more than 2 edges, therefore we need to change the elements "1" in `S` back into 0;
* The states of all the other cells will not be changed by the division of cell `m` and therefore we do not need to update them.

– **Update `N`:**

After each cell division, the algorithm updates the adjacency matrix according to the following observations for each of the five classes:

(a) For daughter cell `m`, the other daughter cell becomes its neighbor, the cells in the class `npneicell` are no longer its neighbors, while all the other neighborhood relationships involving cell `m` remain the same;

(b) For the daughter cell that is put in the tail of `S`, the other daughter cell `m`, the cells in the classes `endcell` and `npneicell` are its neighbors. Accordingly, the algorithm sets the corresponding entries in `N` to 1;

(c) For cells in the class `endcell`, they gain one more neighbor, which is the daughter cell labeled `length(S)`;

(d) For cells in the class `npneicell`, daughter `m` is no longer their neighbor, while the other daughter cell becomes their neighbor;

(e) Finally, all the other neighborhood relationships remain the same.

Recall that the number of experiments denoted by `Batchnumber` and the number of cycles in each experiment denoted by `Cycle` are defined by the user. In the Command Window, during the first experiment, `ccheck` will show how many cycles are completed, and then `batchcheck` will show the number of experiments completed whenever this number is a multiple of 5.
At the end of cell division cycles number 9, 10, and 11, when `Cycle` is greater than 9, 10 or 11 respectively, as well as the end of all the division cycles, the script will call `DataCollection.m` to collect the data we are interested in. Finally, the script will call `DataReport.m` to save and report these data.

2. `DataCollection.m` — This file is called by `AnyOrderCellSides.m` at the end of each individual experiment in a batch. Furthermore, it is also called at the end of cell division cycles 9, 10 and 11 when the user-define parameter `Cycle` for the total number of division cycles is greater than or equal to 9, 10 or 11 respectively. They are then stored under the names specified below followed by "c9", "c10" and "c11" respectively. For example, the total number of cells after 9 cycles' cell division is stored in a vector named `TOTALNUMBERc9`.
Specifically, this script computes the statistics listed below. Note that after a division cycle has been completed, each edge has potentially two orientations. When one orientation is chosen, it uniquely determines a pair (i, j) that signifies the number i of sides of the cell to the left of the edge and the number j of sides of the cell to the right of the edge. Our algorithms mimics random uniform sampling by dividing the counts of all possible pairs by 2.

- `TOTALNUMBER` — A row vector that stores the total number of cells for each experiment.
- `TOTALE` — A row vector that stores the total number of edges shared by real cells for each experiment (as opposed to edges that have a "fake cell" as neighbor).
- `AVEBEFMSIDES` — A row vector that stores the mean number of edges of the cells right before their division for each experiment.
- `STDBEFMSIDES` — A row vector that stores the standard deviation of the polygonal distribution of the cells right before cell division for each experiment.
- `AVEAFTERMSIDES` — A row vector that stores the mean number of edges of the cells right after their division for each experiment.
- `STDAFTERMSIDES` — A row vector that stores the standard deviation of the polygonal distribution of the cells right after cell division for each experiment.

- `MEANSIDEN` — A row vector that stores the mean number of edges of the cells after completion of each experiment.
- `STDEVIATION` — A row vector that stores the standard deviation of the polygonal distribution of the cells after completion of each experiment.
- `DISTRIBUTION4TO9` — A 2D matrix, where each row is a polygonal (from quadrangle to nonagon) distribution of the cells of each experiment.
- `PROP10ORLARGER` — A row vector that stores the proportion of cells with no less than 10 edges for each experiment.
- `MAXEDGENUMBER` — A row vector that stores the maximum number of edges of the cells for each experiment.
- `EDGEDATAWITHIN12` — A 3D matrix that stores a 2D matrix for each experiment, where each 2D matrix shows the number of each type of edges connecting real cells. Specifically, let `EdgeDatawithin12` denote such a 2D matrix,
  then `EdgeDatawithin12(i,j)` is the number of edges with an i-sided cell to the left and a j-sided cell to the right, and the edges for which i or j is greater than 12 are not counted in this matrix.
- `LR` — A 3D matrix that stores a 2D matrix for each experiment, where each 2D matrix shows the number of each type of edges connecting real cells. Specifically, let `lr` denote such a 2D matrix,
  then `lr(i,j)` is the number of edges with an i-sided cell to the left and a j-sided cell to the right.
- `PREDGEWITHIN12` — A 3D matrix, similar to `EDGEDATAWITHIN12`, the only difference is that instead of the number of each type of edges, it stores the proportion of each type of edges that connect real cells relative to all types of edges connecting real cells.
- `PRLR` — A 3D matrix, similar to `LR`, the only difference is that instead of the number of each type of edges, it stores the proportion of each type of edges that connect real cells relative to all types of edges connecting real cells.
- `PREDGE4TO9` — A 3D matrix, where each 2D matrix stored for each experiment is a submatrix of the corresponding 2D matrix in `PREDGEWITHIN12`, which focuses on the edges shared by cells with 4 to 9 edges.
- `PLORPR4TO9` — A 2D matrix, where each row is a probability distribution of the number of sides (from 4 to 9) of the cell to the left (or right) of a randomly chosen edge of each experiment. Here, the outermost edges are not counted.
- `TOTALAE` — A row vector that stores the total number of edges for each experiment, including the outermost edges.
- `PROUTEREDGE` — A row vector that stores the proportion of the outermost edges relative to the total number of edges for each experiment.
- `AEDGEDATAWITHIN12` — A 3D matrix that stores a 2D matrix for each experiment, where each 2D matrix shows the number of each type of edges while the outermost edges are counted. Specifically, let `AEdgeDatawithin12` denote such a 2D matrix, then `AEdgeDatawithin12(1,j)` and `AEdgeDatawithin12(i,1)` where i and j are greater than 1 represent the numbers of the outermost edges with a $j-$sided cell to

the right and the number of the outhermost edges with an $i-$ sided cell to the left respectively. `AEdgeDatawithin12(i,j)` where both i and j are greater than 1 is the number of edges with an i-sided cell to the left and a j-sided cell to the right, and the edges for which i or j is greater than 12 are not counted in this matrix.

- `ALR` — A 3D matrix that stores a 2D matrix for each experiment, where each 2D matrix shows the number of each type of edges while the outermost edges are counted. Specifically, let `Alr` denote such a 2D matrix, then `Alr(1,j)` and `Alr(i,1)` where i and j are greater than 1 represent the numbers of the outermost edges with a $j-$sided cell to the right and the number of the outermost edges with an $i-$ sided cell to the left respectively. `Alr(i,j)` where both i and j are greater than 1 is the number of edges with an i-sided cell to the left and a j-sided cell to the right.
- `PRAEDGEWITHIN12` — A 3D matrix, similar to `AEDGEDATAWITHIN12`, the only difference is that instead of the number of each type of edges, it stores the proportion of each type of edges relative to all types of edges including the outermost ones.
- `PRALR` — A 3D matrix, similar to `ALR`, the only difference is that instead of the number of each type of edges, it stores the proportion of each type of edges relative to all types of edges including the outermost ones.
- `PREDGE0AND4TO9` — A 3D matrix, where each 2D matrix stored for each experiment is a submatrix of the corresponding 2D matrix in `PRAEDGEWITHIN12`, which focuses on the outermost edges and the edges shared by cells with 4 to 9 edges.
- `APLORPR0AND4TO9` — A 2D matrix, where each row is a probability distribution of the number of sides (0 and from 4 to 9) of the cell to the left (or right) of a randomly chosen edge of each experiment. Here, the outermost edges are counted.
- `PRLPRR` — A 3D matrix that stores a 2D matrix for each experiment. Specifically, let `PrlPrr` denote this 2D matrix for an experiment, then for i and j between 1 and 6, `PrlPrr(i,j)` is the product of the probability that an $(i+3)$-sided cell is to the left of a randomly chosen edge and the probability that a $(j+3)$-sided cell is to the right of a randomly chosen edge. Here the outermost edges are not counted;
- `APRLPRR` — A 3D matrix that stores a 2D matrix for each experiment. Specifically, let `APrlPrr` denote this 2D matrix for an experiment, then for i and j between 2 and 7, `APrlPrr(i,j)` is the product of the probability that an $(i+2)$-sided cell is to the left of a randomly chosen edge and the probability that a $(j+2)$-sided cell is to the right of a randomly chosen edge. For i between 2 and 7, `APrlPrr(1,i)` is the product of the probability that a fake cell is to the left of a randomly chosen edge and the probability that an $(i+2)$-sided cell is to the right of a randomly chosen edge, `APrlPrr(i,1)` is the product of the probability that an $(i+2)$-sided cell is to the left of a randomly chosen edge and the probability that a fake cell is to the right of a randomly chosen edge. Here the outermost edges are counted;
- `DIS` — A 3D matrix, where each 2D matrix stored for each experiment measures the discrepancies of the entries in `PrlPrr` and `PrEdge4to9`.
- `ADIS` — A 3D matrix, where each 2D matrix stored for each experiment measures the discrepancies of the entries in `APrlPrr` and `PrEdge0and4to9`.
- `RHO` — A row vector that stores the coefficient of correlation for the random variables representing [the number of sides of the cell to the left of a randomly chosen edge]

and [the number of sides of the cell to the right of a randomly chosen edge]. Here the outermost edges are not counted.

- `ARHO` — A row vector that stores the coefficient of correlation for the random variables representing [the number of sides of the cell to the left of a randomly chosen edge] and [the number of sides of the cell to the right of a randomly chosen edge]. Here the outermost edges are counted.

3. **DataReport.m** — Called by `AnyOrderCellSides.m` at the end of each batch of experiments. Saves the data of interest and displays some of them to the screen.
In order to save our data, it creates a folder named after the parameters that were specified by the user. All the data of interest will be saved in this folder. For example, a folder named `'12CycleRandom-OrthSmpN-Even-Binomial-7-0.65-0.015-0.3'` saves the data of a batch of experiments where

   (a) the number of cell division cycle in each experiment is 12,
   (b) the order of cell division is `'Random'`,
   (c) the strategy of choosing side1 is `'OrthSmpN'`,
   (d) the strategy of choosing side2 is `'Even-Binomial'`,
   (e) the initial state is 7, which means each experiment starts with a 7-sided cell,
   (f) the parameter `smp` is 0.65,
   (g) the standard deviation of the normal distribution is 0.015,
   (h) the parameter `probB` is 0.3.

   The mean values of the data collected by `DataCollection.m` will be displayed in the Command Window automatically when the whole simulation is completed. They are denoted by:

   - `MEANTotalnumber`
   - `MEANTotalE`
   - `MEANAveBefMsides`
   - `MEANStDBefMsides`
   - `MEANAveAfterMsides`
   - `MEANStDAfterMsides`
   - `MEANMeansiden`
   - `MEANStDeviation`
   - `MEANDistribution4to9`
   - `MEANProp10orlarger`
   - `MEANMaxEdgeNumber`
   - `MEANEdgeDatawithin12`
   - `MEANPrEdgewithin12`
   - `MEANPrEdge4to9`
   - `MEANPlorPr4to9`

- `MEANPrlPrr`
- `MEANTotalAE`
- `MEANPrOuterEdge`
- `MEANAEdgeDatawithin12`
- `MEANPrAEdgewithin12`
- `MEANPrEdge0and4to9`
- `MEANAPlorPr0and4to9`
- `MEANAPrlPrr`
- `MEANDis`
- `MEANADis`
- `MEANrho`
- `MEANArho`

Moreover, all theses mean values, the standard deviations of the data collected above in `DataCollection.m`, together with such mean values and standard deviations corresponding to the end of 9, 10 and 11 cell division cycles when the number of division cycles is greater than 9, 10 or 11 respectively, are saved in XLS files. These files are named `means.xlsx`, `stds.xlsx`, `c* means.xlsx` and `c* stds.xlsx`, where $* \in \{9, 10, 11\}$. Finally, all the raw data collected are saved in MAT files `raw data.mat` and `c* raw data.mat` where $* \in \{9, 10, 11\}$. When such a file is created, it will be moved into the folder we created before. After that, this folder will be moved into the folder `Data`.
