# Supplementary material for: Development of epithelial tissues: How are cleavage planes chosen?
Source: PLoS One. 2018 Nov 7;13(11):e0205834. doi: 10.1371/journal.pone.0205834 (PMC6221281; doi:10.1371/journal.pone.0205834)
Supplement: S2 Appendix — (PDF) [file pone.0205834.s002.pdf]

# Simulating the process of cell division with MatLab: A technical lemma

Winfried Just and Ying Xin
Department of Mathematics, Ohio University

**Lemma:** *If $I$, the number of edges between side1 and side2, can never be zero, then at any stage, for any cell $m$, and any two of its edges that do not share a common vertex, the two corresponding neighbor cells cannot be adjacent. Moreover, no two cells can share more than one edge.*

**Proof:** We prove this result by induction over the steps of the simulation, where each step corresponds to the division of a single cell.

At the very beginning of the whole process, we define the initial state and its corresponding neighborhood matrix in a way where each initial real cell has no less than four edges and for a given such cell $m$, if any two edges of $m$ are not adjacent, then its two corresponding neighbor cells are not neighbors of each other. Moreover, we specify the initial states in such a way that no two distinct (real or fake) cells share more than one common edge.

Consider the next step in the algorithm. Let $m$ be the cell that will be divided at this step. Notice that after one cell's division, we will have one more cell $\hat{m}$, which is $m$'s other daughter cell. Changes in the neighborhood relationship can only occur in the following ways:

1. The two cells $m, \hat{m}$ become neighbors and share exactly one common edge.
2. Some cells that were adjacent to $m$ prior to the division step stop being $m$'s neighbors and become $\hat{m}$'s neighbors instead.
3. If an edge $c$ of $m$ is being split up into two edges $c_1, c_2$, then exactly one of them (say, $c_1$) will be an edge *only* of daughter cell $m$, but not of $\hat{m}$ while the other new edge, $c_2$, will be an edge *only* of daughter cell $\hat{m}$, but not of daughter cell $m$.

Notice that the last sentence of the lemma follows immediately by induction from the first and the third of the above observations.

For the proof of the first sentence of the lemma, note that the situation is exactly symmetric for $m$ and $\hat{m}$ after division, so that from now on we may wlog focus on $\hat{m}$ and will use the symbol $m$ mostly for referring to the *mother cell.* Note also that any two edges of $m$ that were already present prior to division will share a common vertex after division if, and only if, they shared a common vertex prior to division.

Now consider any cell $m^*$ and any two edges of it, denoted by edge $a$ and edge $b$. Let us use the symbols $m_a, m_b$ for the two corresponding neighbor cells of $m^*$ across these edges. As

changes of the neighborhood relationship only occurs among $m$'s daughter and neighbor cells, we can restrict our attention to $m^*$ that is one of these cells. We have the following cases:

1. Cell $m^*$ is an "in-between" neighbor cell of $\hat{m}$, that is, a neighbor cell of $\hat{m}$ for which the edge shared by $m^*$ and $m$ was not divided. In this case, for $m^*$ and its neighbors, the only change is that the role of $m$ is replaced by $\hat{m}$. Thus, for any two edges $a$ and $b$ of $m^*$, if they do not share a common vertex, cell $m_a$ and cell $m_b$ were not neighbors prior to the division step and will not become neighbors.

2. Cell $m^*$ is an "endcell," that is, $m^*$ is a neighbor cell of $m$ for which the edge $c$ shared by $m^*$ and $m$ is divided into two edges $c_1, c_2$, where $c_2$ will be shared with $\hat{m}$ after division.

   Now consider two edges $a, b$ of $m^*$ after division.

   (a) If $\{a, b\} \cap \{c_1, c_2\} = \emptyset$, then the same argument as in the previous case applies.

   (b) If $\{a, b\} = \{c_1, c_2\}$, then the two edges share a common vertex and there is nothing to prove.

   (c) $\{a, b\} \cap \{c_1, c_2\}$ has exactly one element. By symmetry, we may assume wlog that $a = c_2$.

   - If $a$ and $c_2$ share a common vertex, there is nothing to prove.
   - If $a, c$ did not share a common vertex, then $m_a, m_b$ were not adjacent prior to division and cannot become adjacent after the division, as the role of $m$ is replaced by its two daughter cells.
   - $a$ and $c$ did share a common vertex $v$, but $a$ and $c_2$ do not. Then prior to division $v$ must have represented a triangular junction of $a, c$ and a common edge $d$ of $m_a$ and $m$. This common edge $d$ cannot have been split up during the current division of $m$ as this would have created a three-sided cell. Therefore, after division, $v$ must represent a triangular junction of $c_1, a, d$, where both $c_1, d$ are edges of daughter cell $m$. Since the two daughter cells $m, \hat{m}$ share only one edge by the part of the lemma that we have already proved, and this one edge must be a newly created one, if $\hat{m} = m_{c_2}$ and $m_a$ were to share an edge, it would need to be (a part of) a common edge of $m_a$ and $m$ prior to division *other than* $d$. This possibility is ruled out though by the part of the lemma that we have already proved.

3. $m^*$ is a daughter cell; wlog $m^* = \hat{m}$. Again consider two edges $a, b$ of $\hat{m}$ after division.

   (a) Both edges $a, b$ are edges of $m$ prior to division. In this case, neither the property of having a common edge nor the neighborhood relationship between $m_a, m_b$ are changed, and it follows from the inductive assumption that if $a, b$ do not have a common vertex, then $m_a, m_b$ will not be adjacent.

   (b) Edge $a$ is the edge shared by the two daugther cells. In this case, if $a$ and $b$ do not share a common vertex, $m_b$ can only be an "in-between cell," more precisely, a cell in the class *npneicell* (since we have assumed wlog that $m^* = \hat{m}$). Our algorithm removes adjacency between cells in this class and daughter cell $m = m_a$, so that $m_a$ and $m_b$ will not be adjacent.

(c) Both edges $a$ and $b$ resulted from splitting up edges $a^+, b^+$ of mother cell $m$. In this case, both cells $m_a$ and cell $m_b$ are "endcells," and edges $a^+, b^+$ cannot have shared a common vertex prior to division, so that $m_a, m_b$ are not adjacent by inductive assumption.

(d) Edge $a$ resulted from splitting an edges $a^+$ of mother cell $m$ while edge $b$ is an edge of mother cell $m$. Then $m_a$ is an "endcell" and $m_b$ is an "in-between cell." Their neighborhood relationship is not changed, and if $a^+$ and $b$ do have a common vertex, this common vertex must be inherited by $a$ since the algorithm assigns contiguous stretches of edges to each daughter cell. Again, it follows from the inductive assumption that if $a, b$ do not have a common vertex, then $m_a, m_b$ will not be adjacent.

□
