# Supplementary material for: Development of epithelial tissues: How are cleavage planes chosen?
Source: PLoS One. 2018 Nov 7;13(11):e0205834. doi: 10.1371/journal.pone.0205834 (PMC6221281; doi:10.1371/journal.pone.0205834)
Supplement: S1 Table — (PDF) [file pone.0205834.s005.pdf]

Complete list of all parameter settings that were explored

Winfried Just and Ying Xin
Department of Mathematics, Ohio University

Pairs and values of the relevant parameters when the division order is set to be '`Random`':

'`Choice1`'	'`Choice2`'	Parameter values (if any involved)
`LaN`	`Binomial`	
`LaN`	`evensplit`	
`OrthBornLaN`	`Binomial`	
`OrthBornLaN`	`evensplit`	
`OrthBornLaN`	`random`	
`OrthBornLaN`	`rotNorm`	`stdbeta` = 0.05, 0.1, 0.15, 0.25, 0.3, 0.4, 0.5, 0.6, 0.7, 0.8, 0.9, 1
`OrthBornLaN`	`rotTanNrom`	`stdbeta` = 0.025, 0.05, 0.075, 0.1, 0.125, 0.25, 0.5, 0.75, 1, 1.5
`OrthBornSmN`	`Binomial`	
`OrthBornSmN`	`evensplit`	
`OrthBornSmN`	`random`	
`OrthBornSmN`	`rotNorm`	`stdbeta` = 0.1, 0.25, 0.3, 0.35, 0.4, 0.45, 0.5, 0.6, 0.7, 0.8, 0.9, 1
`OrthBornSmN`	`rotTanNorm`	`stdbeta` = 0.1, 0.2, 0.225, 0.25, 0.275, 0.3, 0.5, 0.75, 1, 1.25, 1.5
`OrthLaN`	`Binomial`	
`OrthLaN`	`evensplit`	
`OrthLaN`	`random`	
`OrthLaN`	`rotNorm`	`stdbeta` = 0.1, 0.25, 0.3, 0.35, 0.4, 0.45, 0.5, 0.6, 0.7, 0.8, 0.9, 1
`OrthLaN`	`rotTanNorm`	`stdbeta` = 0.0125, 0.025, 0.05, 0.1, 0.25, 0.5, 0.75, 1, 1.25, 1.5
`OrthRandN`	`Binomial`	
`OrthRandN`	`evensplit`	
`OrthRandN`	`random`	
`OrthRandN`	`Even-Binomial`	`probB` = 0.05, 0.1, 0.15, 0.2, 0.25, 0.3, 0.35, 0.4, 0.45, 0.5, 0.55
`OrthRandN`	`rotNorm`	`stdbeta` = 0.025, 0.05, 0.075, 0.1, 0.125, 0.25, 0.3, 0.35, 0.4, 0.45, 0.5, 0.6, 0.7, 0.8, 0.9, 1
`OrthRandN`	`rotTanNorm`	`stdbeta` = 0.0125, 0.025, 0.05, 0.075, 0.1, 0.125, 0.25, 0.5, 0.75, 1

OrthSmN	Binomial	
OrthSmN	evensplit	
OrthSmN	random	
OrthSmN	rotNorm	stdbeta = 0.1, 0.2, 0.225, 0.25, 0.275, 0.3, 0.35, 0.4, 0.45, 0.5, 0.6, 0.7, 0.8, 0.9, 1
OrthSmN	rotTanNorm	stdbeta = 0.1, 0.2, 0.225, 0.25, 0.275, 0.3, 0.5, 0.75, 1, 1.25, 1.5
OrthSmpN	Even-Binomial	smp = 0.525, 0.55, 0.6<br>probB = 0.05, 0.1, 0.15, 0.2, 0.25, 0.3, 0.35, 0.4, 0.45, 0.5, 0.55
OrthSmpN	evensplit	smp = 0.2, 0.25, 0.3, 0.35, 0.4, 0.45, 0.475, 0.525, 0.55, 0.6, 0.625, 0.65, 0.675, 0.7
OrthSmpN	rotNorm	smp = 0.2, 0.25, 0.3, 0.35, 0.4, 0.45, 0.475, 0.525, 0.55, 0.6, 0.65, 0.7<br>stdbeta = 0.0125, 0.025, 0.0375, 0.05, 0.0625, 0.075, 0.0875, 0.1, 0.125, 0.15, 0.2, 0.25, 0.5
OrthSmpN	rotTanNorm	smp = 0.2, 0.25, 0.3, 0.35, 0.4, 0.45, 0.475, 0.525, 0.55, 0.6, 0.65, 0.7<br>stdbeta = 0.0125, 0.025, 0.0375, 0.05, 0.0625, 0.075, 0.0875, 0.1, 0.125, 0.15, 0.2, 0.25, 0.5
RandN	Binomial	
RandN	evensplit	
RandN	random	
SmN	Binomial	
SmN	evensplit	
SmN	random	
SmN	Even-Binomial	probB = 0.7, 0.75, 0.8, 0.85
SmN	rotNorm	stdbeta = 0.1, 0.225, 0.25, 0.275, 0.3, 0.35, 0.4, 0.45, 0.5, 0.6, 0.7, 0.8, 0.9,1
SmN	rotTanNorm	stdbeta = 0.1, 0.125, 0.15, 0.175, 0.2, 0.225, 0.25, 0.275, 0.3, 0.325, 0.35, 0.375, 0.4, 0.425, 0.45, 0.475, 0.5, 0.525, 0.55, 0.575, 0.6, 0.625, 0.65, 0.675, 0.7, 0.725, 0.75, 1, 1.25, 1.5

The 'Choice1'|'Choice2' pairs are chosen based on similar pairs chosen in [2] and predictions made in [1] and [2]. The parameter values are chosen as we see appropriate while running the simulations, with a relatively wide range set up beforehand.

Pairs and values of the relevant parameters when the division order is set to be ‘`Strict`’ are the same as above, except that in this case, the following settings are not included:

‘Choice1’	‘Choice2’	Parameter values (if any involved)
`OrthRandN`	`Even-Binomial`	`probB` = 0.05, 0.1, 0.15, 0.2, 0.25, 0.3, 0.35, 0.4, 0.45, 0.5, 0.55
`OrthSmpN`	`Even-Binomial`	`smp` = 0.525, 0.55, 0.6<br>`probB` = 0.05, 0.1, 0.15, 0.2, 0.25, 0.3, 0.35, 0.4, 0.45, 0.5, 0.55

This is because such options are included for the case of division order being `‘Random’` only after all other simulations are done and sorted according to the χ^2-statistics, for the purpose of a comparison between `‘Even-Binomial’` and our front-runners with `‘Choice2’` being `‘rotNorm’` or `‘rotTanNorm’`.

References

[1] Gibson MC, Patel AB, Nagpal R, Perrimon N. Emergence of geometric order in proliferating epithelia. Nature. 2006; 442:1038–1041.

[2] Patel AB, Gibson WT, Gibson MC, Nagpal R. Modeling and Inferring Cleavage Patterns in Proliferating Epithelia. PLoS Comput. Biol. 2009; 5(6), e1000412.
